# Molecular mechanism for the substrate recognition of USP7

**DOI:** 10.1007/s13238-015-0192-y

**Published:** 2015-07-26

**Authors:** Jingdong Cheng, Ze Li, Rui Gong, Jian Fang, Yi Yang, Chang Sun, Huirong Yang, Yanhui Xu

**Affiliations:** Key Laboratory of Molecular Medicine, Ministry of Education, Department of Systems Biology for Medicine, School of Basic Medical Sciences, Shanghai Medical College of Fudan University, Shanghai, 200032 China; School of Basic Medical Sciences, Fudan University Shanghai Cancer Center, Institutes of Biomedical Sciences, Shanghai Medical College of Fudan University, Shanghai, 200032 China; State Key Laboratory of Genetic Engineering, School of Life Sciences, Collaborative Innovation Center of Genetics and Development, Fudan University, Shanghai, 200433 China

**Dear Editor,**

The ubiquitin specific protease 7 (USP7), also known as herpes virus associated ubiquitin specific protease (HAUSP), is a well-characterized deubiquitinase (Reyes-Turcu et al., 2009). USP7 plays important roles in various biological processes, including cell survival, proliferation, apoptosis, tumorigenesis, viral infection, and epigenetic regulation through regulating the protein stability of tumor suppressors (p53, PTEN, FOXO, claspin), E3 ligases (MDM2, Mule, viral proteins ICP0), epigenetic regulators (DNMT1, Tip60, UHRF1) (Pfoh et al., 2015). USP7 is comprised of three recognizable domains: the N-terminal TRAF domain, the catalytic domain, and the C-terminal Tandem UBL domain (designated TUD^USP7^) (Fig. 1A). Previous study shows that the TRAF domain binds to P/AxxS motif (from p53 and MDM2) and is responsible for substrate recognition (Sheng et al., 2006). However, how USP7 recognizes other substrates remains largely unknown.

**Figure 1:**
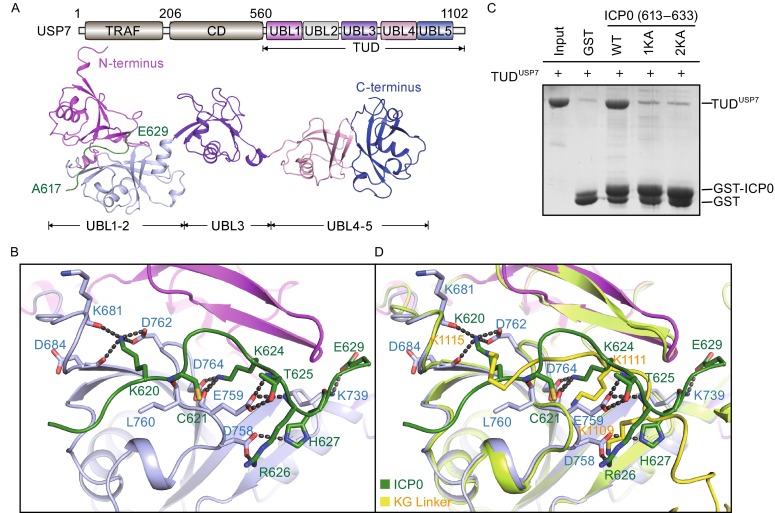
**Crystal structure of the USP7-ICP0 complex**. (A) Color-coded domain architecture of human USP7 (top) and ribbon representations of the overall structure of the TUD^USP7^-ICP0 complex (bottom). The color scheme is used in all structural figures. The ICP0 peptide is colored in forest. The N- and C-termini of USP7 and ICP0 are indicated. (B) Closed-up view of the intermolecular interaction. USP7 and ICP0 are shown in ribbon representations. Critical residues for the interaction are shown in stick representation. Hydrogen bonds are shown as dashed lines. (C) GST pull-down assay for the interaction between TUD^USP7^ and ICP0. Wild type and mutants of GST-tagged ICP0 (613–633) were incubated with TUD^USP7^ and immobilized on glutathione resin. The bound proteins were subjected to SDS-PAGE and visualized by Coomassie blue staining. The free GST is resulted from protein purification and would not affect the results. (D) Comparison of TUD^USP7^-ICP0 and USP7-DNMT1 structures. In USP7-DNMT1 structure, USP7 is colored in lemon and the KG-Linker in yellow. Critical residues for the interactions are shown in stick representation. Hydrogen bonds are shown as dashed lines

Previous study shows that TUD^USP7^ is important for the regulation of USP7 enzymatic activity (Faesen et al., 2011). We have previously showed that USP7 stabilizes UHRF1 through the interaction between TUD^USP7^ and a Spacer region between the SRA and RING domain of UHRF1 (Ma et al., 2012). Our recent study indicates that USP7 binds to and stabilizes DNMT1 through the interaction between an acidic pocket of TUD^USP7^ and the KG repeat region (KG-Linker) of DNMT1. We therefore speculate that TUD^USP7^ may function as a binding site for some other USP7-binding proteins or substrates. To test this hypothesis, we first investigated the interaction between USP7 and the Herpes simplex virus type 1 (HSV1) regulatory protein ICP0 (also known as Vmw110), which is the first identified USP7-interacting protein and regulates the lytic and latent viral infection through inducing proteasomal degradation of various cellular proteins (Boutell and Everett, 2013).

It has been shown that ICP0 peptide (residues 594–633) interacts with the TUD of USP7 (Everett et al., 1999). To investigate how ICP0 binds to TUD^USP7^, we determined the crystal structure of TUD^USP7^ in complex with ICP0 (613–633) at 2.70 Å resolution (Fig. 1A, Table S1). In the TUD^USP7^-ICP0 complex structure, the TUD^USP7^ adopts an extended conformation, and shares similar fold with the TUD^USP7^ alone (2YLM.PDB) (Faesen et al., 2011) (Fig. S1). As observed in previous studies (Faesen et al., 2011; Cheng et al., 2015), UBL1–2, UBL3, and UBL4–5 of TUD^USP7^ form three separate modules and are connected by flexible regions flanking the UBL3. The ICP0 packs against an acidic groove on the surface of UBL1–2. Most of the residues involved in intermolecular interaction are well covered by the electron density, indicating the model was correctly built (Fig. S2).

As indicated in the TUD^USP7^-ICP0 structure, the interaction is mediated by a network of hydrogen bonds and salt bridge contacts. Residue K620^ICP0^ forms hydrogen bonds with the carbonyl oxygen atoms of residues K681^USP7^, D684^USP7^ and side chain of D762^USP7^. Residue K624^ICP0^ snugly inserts into an acidic cavity, with the side chain stabilized by residues E759^USP7^ and D764^USP7^. Hydroxyl group of T625^ICP0^ forms hydrogen bond with E759^USP7^. Residues R626^ICP0^ and H627^ICP0^ form hydrogen bonds with side chain of residue D758^USP7^. The carboxyl group of residue E629 interacts with the side chain of K739^USP7^. The main chains of ICP0 (residues C621, T625, and R626) form hydrogen bonds with residue K739^USP7^, L760^USP7^, and E759^USP7^ (Fig. 1B). Consistent with the observation from the structural analyses, mutations K624A^ICP0^ (1KA) and K620A/K624A^ICP0^ (2KA) of ICP0, largely decreased the binding affinities to TUD^USP7^, compared with the wild-type ICP0 (Fig. 1C), supporting the key role of these residues in mediating the intermolecular interaction.

Our previous study indicates that the KG-Linker of DNMT1 binds to TUD^USP7^ and plays an important role in mediating DNMT1-USP7 interaction and USP7-mediated stabilization of DNMT1. Structural comparison of USP7-DNMT1 and USP7-ICP0 indicates that ICP0 and KG-Linker^DNMT1^ pack against the same acidic pocket of TUD^USP7^ in an opposite orientation (Cheng et al., 2015). As shown in Fig. 1D, residues K624^ICP0^ and K1111^DNMT1^ both bind to the acidic cavity formed by residues E736^USP7^, D758^USP7^, E759^USP7^, and D764^USP7^, and residues K620^ICP0^ and K1115^DNMT1^ form hydrogen bonds with D684^USP7^ and D762^USP7^. In support of above observation, mutation D758A/E759A/E764A of TUD^USP7^ (designated M1^USP7-TUD^) largely reduced the binding affinity to ICP0 and DNMT1 peptides in GST pull down experiment (Fig. 2A). The above analyses suggest that TUD^USP7^ may recognize consensus primary sequence containing KxxxK and therefore the acidic pocket of TUD^USP7^ serves as another binding site (besides the TRAF) for recognition of USP7-interacting proteins.

**Figure 2:**
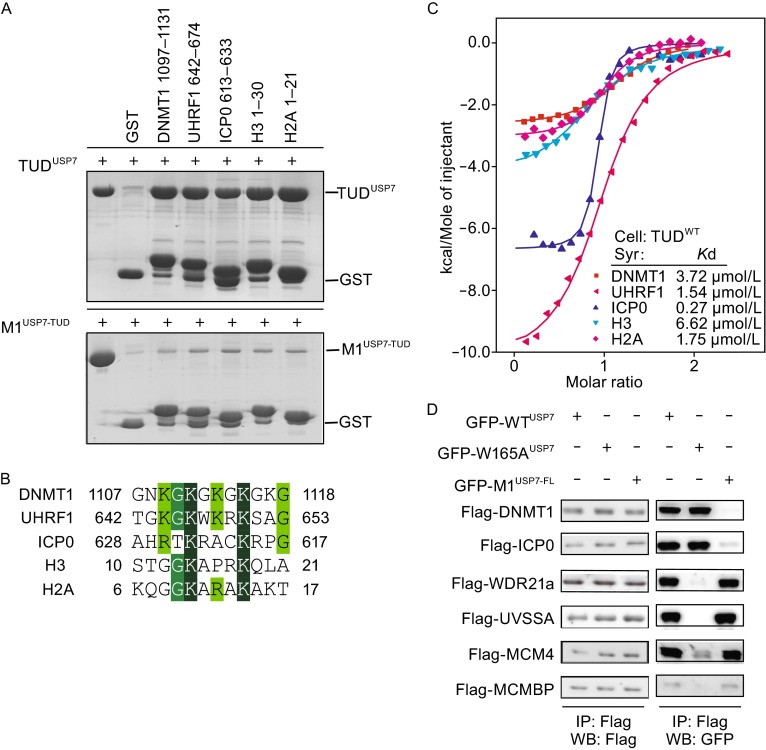
**TUD is a new substrate-binding domain**. (A) DNMT1, UHRF1, ICP0, histone H3 and H2A bind to the same acidic pocket of TUD^USP7^. The GST pull down assay were performed as in Fig. 1C. (B) The alignment of the corresponding sequences of DNMT1, UHRF1, ICP0, H3, and H2A. Identical and highly conserved residues are highlighted in dark green and conserved residues in light green. This indicated that all these protein have the conserved KxxxK motif. (C) ITC measurement of the interactions between TUD^USP7^ and various peptides. (D) The interactions between full-length USP7 and its binding proteins. HEK293T cells were transiently co-transfected with FLAG-tagged proteins and GFP-tagged USP7 (wild-type and mutants) followed by immunoprecipitation. The proteins were detected by immunoblotting using the indicated antibodies

To test the hypothesis, we searched human proteins database and found a number of candidates (including UHRF1, histone H3, histone H2A) contain such consensus motif and potentially bind to TUD^USP7^ (Fig. 2B). We therefore used TUD^USP7^ and M1^USP7-TUD^ (defective binding to KxxxK motif) to investigate that whether these proteins bind to USP7 on the same site. As shown in the GST pull down assays, wild type TUD^USP7^ binds to the KG-Linker of DNMT1, ICP0 (613–633), UHRF1 (642–674), histone H3 (1–30), and histone H2A (1–21) (Fig. 2A). We also confirmed the interaction using the ITC measurement (Fig. 2C). In contrast, mutation of the residues within the acidic pocket (M1^USP7-TUD^) largely decreased the binding affinity to all the five peptides (Fig. 2A). In support of this observation, USP7 has been shown to bind to and stabilize UHRF1 and this interaction is regulated by phosphorylation of UHRF1 during cell cycle (Ma et al., 2012). However, whether histones H3 and H2A are regulated by USP7 *in vivo* needs further investigation.

USP7 is one of the best-characterized deubiquitinases (Nicholson and Suresh Kumar, 2011). Previous studies have identified a number of USP7-binding proteins, including several substrates for deubiquitination (Sowa et al., 2009). Mutation W165A^USP7^ on the TRAF domain has been shown to impair its interaction with p53 and MDM2 (Sheng et al., 2006). We next verified the interaction between USP7 and its binding proteins in cells using co-immunoprecipitation to investigate which site (TRAF or the acidic pocket on TUD^USP7^) on USP7 is responsible for the interaction with distinct binding proteins. Consistent with above analyses, M1^USP7-FL^ abolished its interaction with DNMT1 and ICP0 (Fig. 2D). In contrast, WDR21a, UVSSA, MCMBP, and MCM4 showed comparable USP7-binding affinity to M1^USP7-FL^, but impaired the interaction with W165A^USP7^ (Fig. 2D), indicating that these proteins mainly bind to USP7 on the TRAF domain. Taken together, USP7 binds to distinct proteins on two separate sites, TRAF and the acidic pocket on TUD^USP7^, respectively.

Intriguingly, WDR21a, UVSSA, MCMBP, MCM4, p53, and MDM2 (binds to TRAF domain) are involved in cell cycle, DNA repair or DNA replication, whereas DNMT1 and UHRF1 (binds to TUD^USP7^) are involved in the maintenance of DNA methylation. Thus, our study indicates that the existence of two substrate-binding sties allows USP7 to recognize distinct groups of proteins/substrates for the regulations of different biological pathways. The proteins that bind to the same substrate-binding site may compete with each other for regulation if they exist in the same cellular compartment. Our study also provides a structural basis for designing inhibitors of USP7 that specifically regulates the maintenance of DNA methylation.

## Electronic supplementary material

Supplementary material 1 (PDF 302 kb)
