# Supplementary material for: Molecular mechanism for the substrate recognition of USP7
Source: Protein Cell. 2015 Jul 26;6(11):849–52. doi: 10.1007/s13238-015-0192-y (PMC4624675; doi:10.1007/s13238-015-0192-y)
Supplement: Supplementary file 1 — Supplementary material 1 (PDF 302 kb) [file 13238_2015_192_MOESM1_ESM.pdf]

Supplemental information:

Materials and Methods

Plasmids and Antibodies

The cDNA of WDR21a, UVSSA, MCM4 and MCMBP are provided by Jiahuai Han lab in Xiamen University, The cDNA of ICP0 is provided by Roger D. Everett in University of Glasgow. WDR21a, UVSSA, MCM4 and MCMBP were cloned into pCMV-Tag2b vectors. ICP0 and mutants were cloned into modified pGEX 6p-1 and pCMV-Tag2b. DNMT1, UHRF1, H3 and H2A peptides wre cloned into modified pGEX 6p-1 vector.

The following antibodies were used: FLAG (Abmart, China) and GFP (abclonal technology, China).

Protein Purification

Human USP7 and its constructs were sub-cloned into a modified pGEX-6P-1 plasmid and expressed in the *E. coli* strain BL21 (DE3). The proteins were initially purified using Ni-NTA affinity chromatography, followed by on-column cleavage at 4°C overnight. The proteins were further purified using anion exchange and size exclusion chromatography. The final buffer condition was 20mM Tris-HCl, pH 8.0, 300mM NaCl, and 5mM dithiothreitol (DTT). The peak fractions were collected and concentrated to 10mg/ml for crystallization and biochemical analyses.

DNMT1, UHRF1, H3 and H2A peptide were cloned into modified pGEX 6p-1 vector and been purified following the similar procedure. In differently, there is no on-column cleavage when the peptides were purified for GST pull down assay.

ICP0 peptide was purified following the above procedure. We also synthesized it for crystallization.

Crystallization and Data Collection

Crystals of the USP7-ICP0 complex were grown at 4°C using the hanging drop vapor diffusion method by mixing equal volumes of the purified protein complex and crystallization buffer containing 10% PEG 3350, 100mM Magnesium Sulfate, and 500mM Bis-Tris, pH 6.0. Crystals were equilibrated with a cryoprotectant buffer containing reservoir buffer and additional 20% glycerol (v/v) and were flash frozen in a cold nitrogen stream at -173°C. All data were collected in 0.97852Å wavelength at beamline BL17U at SSRF (Shanghai Synchrotron Radiation Facility, China). The data were processed using the program HKL2000(Otwinowski and Minor, 1997).

Structure Determination

The phase was determined by molecular replacement using the program PHASER(McCoy et al., 2005) with the crystal structures of USP7 (residues 560-1084) (PDB ID: 2YLM) used as search models. The final model was manually built using Coot(Emsley and Cowtan, 2004) . All refinements were performed using the refinement module phenix.refine of the PHENIX package(Adams et al., 2002).

The model quality was validated using the PROCHECK program(Laskowski et al., 1993) , which indicated good stereochemistry according to the Ramachandran plot for the structure. All structural figures were generated using PyMOL (http://www.pymol.org)(DeLano, 2002).

GST Pull-down Assays

Different kinds of ICP0, UHRF1, DNMT1, H3 and H2A peptide with GST tag were incubated with USP7 proteins in binding buffer containing 25mM HEPES, pH 7.4, 150mM NaCl, 5% glycerol, 0.05% Triton X-100, and 1mM DTT for 2 hours at 4°C. The protein samples were then immobilized on 25μl of glutathione resin (GE Healthcare) for 20 minutes at 4°C. The resin was washed three times with binding buffer, and bound proteins were subjected to SDS-PAGE and stained with Coomassie brilliant blue.

Isothermal Titration Calorimetry (ITC)

To obtain the binding affinity between different kinds of peptides with USP7 protein, 0.05mM wild-type USP7 (residues 560-1102) was titrated with 0.5mM different kinds of peptides using iTC200 microcalorimeter (GE healthcare) at 18°C. Both proteins were prepared in a buffer containing 10mM HEPES, pH8.0, and 100mM NaCl. The data were fitted by software Origin 7.0.

Cell culture and transfection

HEK 293T cell was obtained from the American Type Culture Collection and was maintained in Dulbecco's modified Eagle's medium (DMEM, Sigma) containing 10% fetal bovine serum (FBS; Biological Industries, Israel) and supplemented with 2mM glutamine, 100U/mL penicillin, and 100μg/mL streptomycin. Plasmid transfection was performed using linear polyethylenimines (PEIs) (Polysciences).

Immunoprecipitation

The HEK 293T cells were transfected with different kinds of Flag tagged proteins, and cells were harvested 48 hours after transfection. Cells were lysed in RIPA (radioimmunoprecipitation assay) buffer containing a complete protease inhibitor mixture and incubated with anti-FLAG M2 beads (Sigma-Aldrich) for 2 hours at 4°C. The beads were washed 5 times with washing buffer consisting of 20mM Tris-HCl, pH 7.9, 300mM NaCl, 5% glycerol, 0.1% NP-40, and 0.2mM EDTA. The bound proteins were used for assays or immunoblotting.

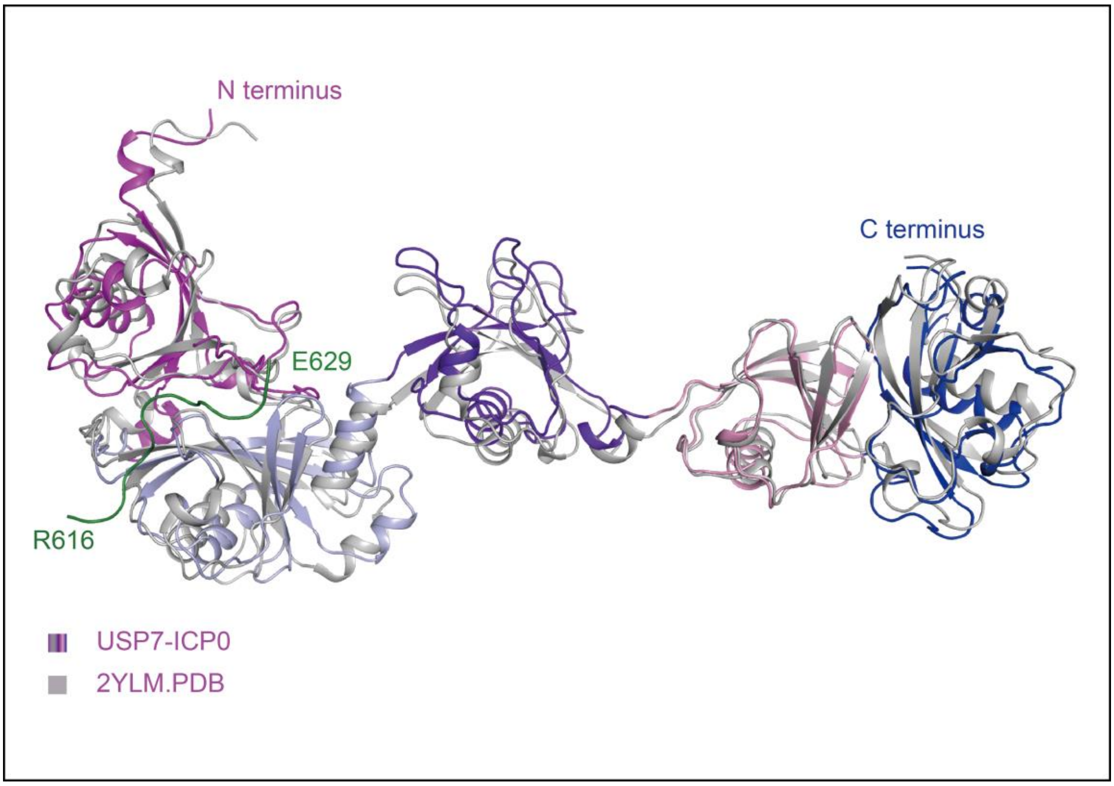

Supplementary Figure S1. Structural comparison of TUD^{USP7}-ICP0 and TUD^{USP7}

Superposition of the TUD^{USP7}-ICP0 and TUD^{USP7} (PDB ID: 2YLM) structures. TUD^{USP7}-ICP0 and TUD^{USP7} structures are shown in ribbon representation. TUD^{USP7}-ICP0 was colored as in Figure 1 and TUD only was colored in grey. The N- and C-termini of USP7 and ICP0 are indicated.

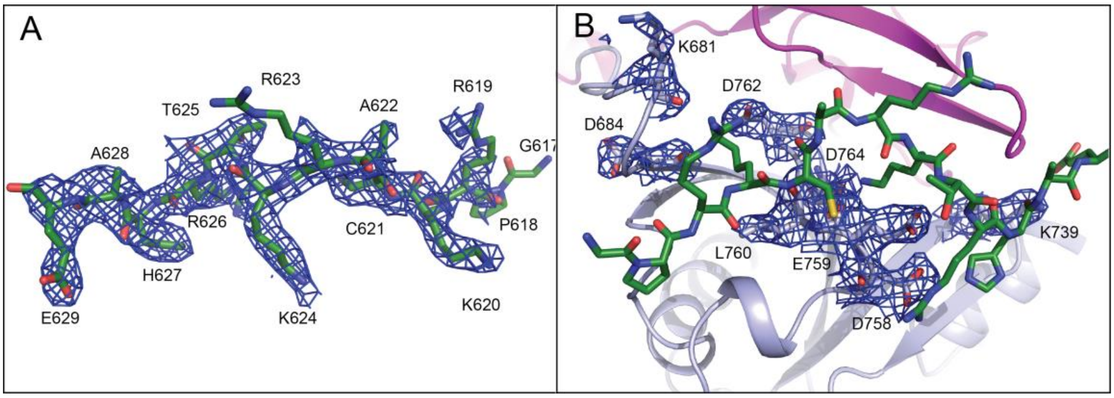

Supplementary Figure S2. Omit map of ICP0 peptide

The 2Fo-Fc Omit map of ICP0 peptide in TUD^{USP7}-ICP0 structure is shown in blue mesh. The isolated ICP0 peptide (A) and the critical residues for ICP0-interaction in USP7 (B) were shown in sticks. The maps were calculated at 2.70 Å, and contoured at 1.0σ. Most of the critical residues are well covered by the electron density.

Supplementary Table 1. Data collection and refinement statistics

	TUD^{USP7}-ICP0
**Data collection**	
Space group	$P2_12_12_1$
Cell dimensions	
a, *b*, *c* (Å)	78.88, 80.38, 151.15
α, β, γ (°)	90, 90, 90
Resolution (Å)	42.46-2.69 (2.781-2.69)*
R_{sym} or R_{merge}	9.6 (86.6)
I /σI	11.11 (2.39)
Completeness (%)	99.31 (94.98)
Redundancy	6.1 (6.2)
**Refinement**	
Resolution (Å)	42.46-2.69 (2.781-2.69)
No. reflections	27339
R_{work} / R_{free}	21.55/26.30
No. atoms	4446
Protein	4440
Water	6
B-factors	
Protein	76.30
Water	53.40
R.m.s. deviations	
Bond lengths (Å)	0.010
Bond angles (°)	1.48

Data set were collected with one native crystal.

*Values in parentheses are for highest-resolution shell.

Reference

Adams, P.D., Grosse-Kunstleve, R.W., Hung, L.W., Ioerger, T.R., McCoy, A.J., Moriarty, N.W., Read, R.J., Sacchettini, J.C., Sauter, N.K., and Terwilliger, T.C. (2002). PHENIX: building new software for automated crystallographic structure determination. Acta Crystallographica Section D-Biological Crystallography 58, 1948-1954.

DeLano, W.L. (2002). The PyMOL Molecular Graphics System.

Emsley, P., and Cowtan, K. (2004). Coot: model-building tools for molecular graphics. Acta Crystallographica Section D-Biological Crystallography 60, 2126-2132.

Laskowski, R.A., Macarthur, M.W., Moss, D.S., and Thornton, J.M. (1993). Procheck - a Program to Check the Stereochemical Quality of Protein Structures. Journal of Applied Crystallography 26, 283-291.

McCoy, A.J., Grosse-Kunstleve, R.W., Storoni, L.C., and Read, R.J. (2005). Likelihood-enhanced fast translation functions. Acta Crystallogr D Biol Crystallogr 61, 458-464.

Otwinowski, Z., and Minor, W. (1997). Processing of X-ray diffraction data collected in oscillation mode. Macromolecular Crystallography, Pt A 276, 307-326.
